# First principle study of scandium-based novel ternary half Heusler ScXGe (X = Mn and Fe) alloys: insight into the spin-polarized structural, electronic, and magnetic properties†

**DOI:** 10.1039/d4ra00811a

**Published:** 2024-04-25

**Authors:** Hayat Ullah, Sadia Yasin, Kashif Safeen, Adeel Younus, Zeinhom M. El-Bahy, Akif Safeen, Safaa N. Abdou, Mohamed M. Ibrahim

**Affiliations:** a Material Modeling and Simulation Lab, Department of Physics, Women University of Azad Jammu & Kashmir Bagh Pakistan hayatullahphys@wuajk.edu.pk; b Department of Physics, Abdul Wali Khan University Mardan 23200 Pakistan kashifsafeen@awkum.edu.pk; c Department of Chemistry, Faculty of Science, Al-Azhar University Nasr City 11884 Cairo Egypt; d Department of Physics, University of Poonch Rawalakot, AJK 12350 Pakistan akifsafeen@upr.edu.pk; e Department of Chemistry, Khurmah University College, Taif University Taif Saudi Arabia; f Department of Chemistry, College of Science, Taif University P. O. Box 11099 Taif 21944 Saudi Arabia

## Abstract

The structural, electronic, and magnetic properties of novel half-Heusler alloys ScXGe (X = Mn, Fe) are investigated using the first principle full potential linearized augmented plane wave approach based on density functional theory (DFT). To attain the desired outcomes, we employed the exchange–correlation frameworks, specifically the local density approximation in combination with Perdew, Burke, and Ernzerhof's generalized gradient approximation plus the Hubbard *U* parameter method (GGA + *U*) to highlight the strong exchange–correlation interaction in these alloys. The structural parameter optimizations, whether ferromagnetic (FM) or nonmagnetic (NM), reveal that all ScXGe (where X = Mn, Fe) Heusler alloys attain their lowest ground state energy during FM optimization. The examination of the electronic properties of these alloys reveals their metallic character in both the spin-up and spin-down channels. The projected densities of states indicate that bonding is achieved through the hybridization of p–d and d–d states in all of the compounds. The investigation of the magnetic properties in ScXGe (where X = Mn, Fe) compounds indicates pronounced stability in their ferromagnetic state. Notably, the Curie temperatures for ScXGe (X = Mn, Fe) are determined to be 2177.02 K and 1656.09 K, respectively. The observation of metallic behavior and the strong ferromagnetic characteristics in ScXGe (X = Mn, Fe) half-Heusler alloys underscores their potential significance in the realm of spintronic devices. Consequently, our study serves as a robust foundation for subsequent experimental validation.

## Abstract 2

Total and partial DOS of non-degenerate d_eg_ and d_t2g_ states of (a) ScMnGe and (b) ScFeGe Heusler alloys.

## Graphical abstract

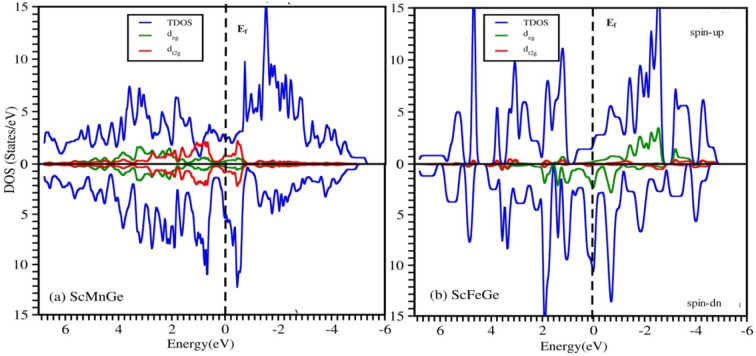

## 1. Introduction

Heusler alloys, originally conceived by Friedrich Heusler in 1903,^1^ have recently garnered significant attention within the scientific community due to their promising potential in the realm of spintronics and smart materials.^2^ Among these alloys, Half-Heusler (HH) semiconductors stand out, characterized by having either eight (08) or eighteen (18) valence electrons and band gaps spanning from 0 to 4 eV. Remarkably, this category encompasses around 250 ternary compounds. Recent research reports have also revealed a multitude of physical phenomena associated with these Heusler alloys, including ferroelectricity, ferromagnetism, and ferroelasticity, attributed largely to their multifunctional properties. As a result, these alloys are continually drawing significant interest in a wide range of fields, including spintronics,^3,4^ optoelectronics (such as sensors, magnetoresistors, photovoltaic detectors, and light-emitting diodes), thermoelectronics,^5,6^ shape memory applications,^2,7^ piezoelectric semiconductors,^3,8^ topological insulators,^4,9^ and superconductivity.^10,11^ Additionally, they offer distinct advantages over conventional electronic devices based on standard semiconductors, owing to the added spin degree of freedom, resulting in benefits such as enhanced data processing speed, increased integration densities, and reduced power consumption.^12–14^ Moreover, the pursuit of achieving fully spin-polarized currents has generated considerable interest in these materials.^15^ Heusler alloys possess another remarkable feature, stemming from their utilization of cost-effective raw materials and their ability to withstand chemical and mechanical stresses at high temperatures and densities. In the realm of thermoelectric applications, Heusler alloys have been subject to extensive investigation.^16^ Therefore, it is of paramount importance to delve into the magnetic, electronic, and structural properties of Heusler alloys. Such exploration not only promises to enhance the efficiency of thermoelectric devices but also offers insights into various underlying physical phenomena associated with their multifunctional properties.

Heusler alloys exhibit a diverse range of crystal structures and are renowned for their distinctive classification. The majority of these crystals adopt a closely packed cubic structure, with four equidistant points within the FCC lattice forming the basis for the unit cell's diagonal.^17^ Heusler alloys are a class of intermetallic compounds that can be categorized into two main groups: ternary and quaternary. Among ternary intermetallic compounds, the principal families consist of full Heusler alloys (X_2_YZ) and half or semi-Heusler alloys (XYZ). Their crystal structures are denoted as L21 and C1b, respectively. Half Heusler alloys are particularly noteworthy for their cost-effectiveness, lightweight nature, and eco-friendly attributes. The typical composition of half Heusler alloys is described as XYZ, where X and Y represent metals of the transition group, with Y being less electronegative, possibly belonging to the alkaline earth metal or rare earth metal group, and Z representing an s–p or main group element.

Extensive attention has been paid to the study of Heusler alloys from both an applied and basic standpoint. The search for novel magnetic materials with high spin polarization is becoming more and more important in order to improve the functionality of spintronic devices, such as spin filters and spin valves. Half-metallic Heusler compounds, which have 100% spin-polarized charge carriers at the Fermi level, are among the most promising prospects for reaching this high spin polarization. Continuously emerging properties and potential applications contribute to the evolving landscape of research in this field. A recent noteworthy development is the anticipation of half-metallic ferromagnetism. Remarkably, the features of many Heusler compounds can be easily predicted based on their valence electron count. In today's technological landscape, magnetic materials play a crucial role, finding applications in diverse areas such as data storage, energy conversion, and contactless sensing.^18^ However, the process of developing new high-performance magnets is both time-consuming and often unpredictable, with only a limited number of magnets gaining widespread adoption in mainstream applications.

Previous research by Karna *et al.*^19^ shown that structural, magnetic, thermodynamic, and charge transport parameters reveal anisotropic metallic behavior in non-centrosymmetric hexagonal ScFeGe, which is characterized by a weak itinerant incommensurate helimagnetic state below *T*_N_ = 36 K. A temperature and field-independent helical wave vector *k* = (0 0 0.193) with magnetic moments of 0.53 bohr magnetons per formula unit were found in the neutron diffraction experiments, which is primarily confined to the *ab*-plane. We have examined the structural, electrical, and magnetic characteristics of ScXGe (where X = Mn, Fe) in this study. A thorough first-principle investigation has not yet been documented, despite the fact that there have only been a few experimental studies into the structural, thermodynamic, magnetic, and charge transport features of ScFeGe. Furthermore, no theoretical or experimental research has examined the structural, electrical, and magnetic properties of ScMnGe using DFT calculations, motivating us to carry out this investigation and contribute to closing the knowledge gap. Our study also elucidates the interesting effects on the structural properties of these compounds of substituting a Fe atom for the Mn atom.

## 2. Computational details

**Fig. 1:**
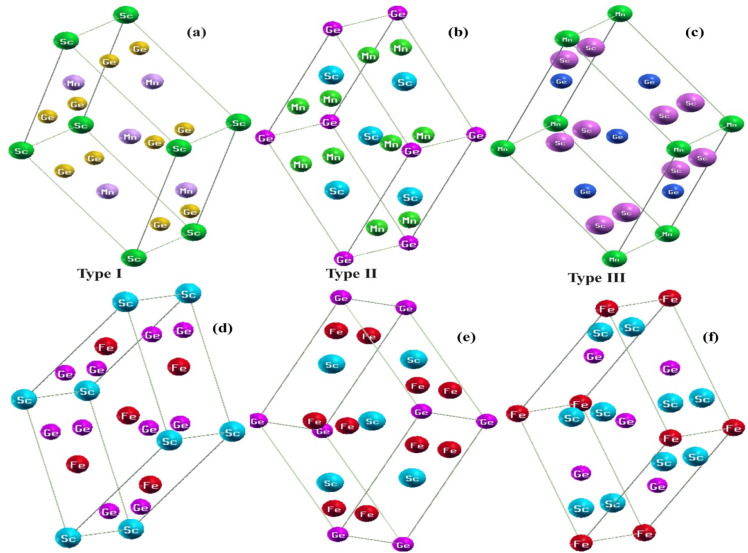
Type I (a and d), type II (b and e) and type III (c and f) crystal structures of ScXGe (X = Mn, Fe) alloys respectively.

The present calculation of structural, electronic, and magnetic properties of scandium-based half Heusler alloys ScXGe (X = Mn, Fe) was performed using the full potential linearized augmented plane wave plus local orbitals (FPLAPW + LO) method based on the density functional theory (DFT) as executed in the Wien2k code. ScXGe (X = Mn, Fe) half Heusler compounds exist in hexagonal structure with space group no. 189 (*P*6̄2*m*). As shown in Fig. 1, the Sc atom is located in a unit cell at location (0.25, 0.25, 0.25) while the Mn and Fe atoms are located at positions (0.5, 0.5, 0.5) and (0 0 0) respectively. For the treatment of exchange–correlation potentials of ScXGe (X = Mn, Fe), we have employed the LSDA, WC-GGA, and PBE-GGA functional. Moreover, the GGA + *U* potential, where *U* is the Hubbard parameter was also added for the treatment of the d state of Mn and Fe to better understand the electronic nature and magnetic structure of ScXGe (X = Mn, Fe). The *U* parameters within the GGA + *U* framework were fine-tuned following the methodology outlined in our previous research.^20^ The *U* parameters in the GGA + *U* were adopted in the range from 7 to 7.9 eV by the method introduced in ref. 21. In these calculations, we have utilized a parameter denoted as *R*_MT_ × *K*_max_ = 7, which specifies the matrix size for convergence. Here, *K*_max_ represents the plane wave cutoff, and the choice of muffin-tin radii (*R*_MT_) is made to ensure that there is no charge leakage from the spherical regions while minimizing the interstitial space. The parameters *G*_max_ and *Ɩ*_max_ (angular momentum vector) are taken as 12 and 6 respectively. We have used 1000 *k* points inside the irreducible first Brillion zone to ensure excellent convergence of the total energy. For the division of valence and core states, the cutoff energy is chosen to be −7.0 Ry.

## 3. Results and discussion

### 3.1 Stability

**Fig. 2:**
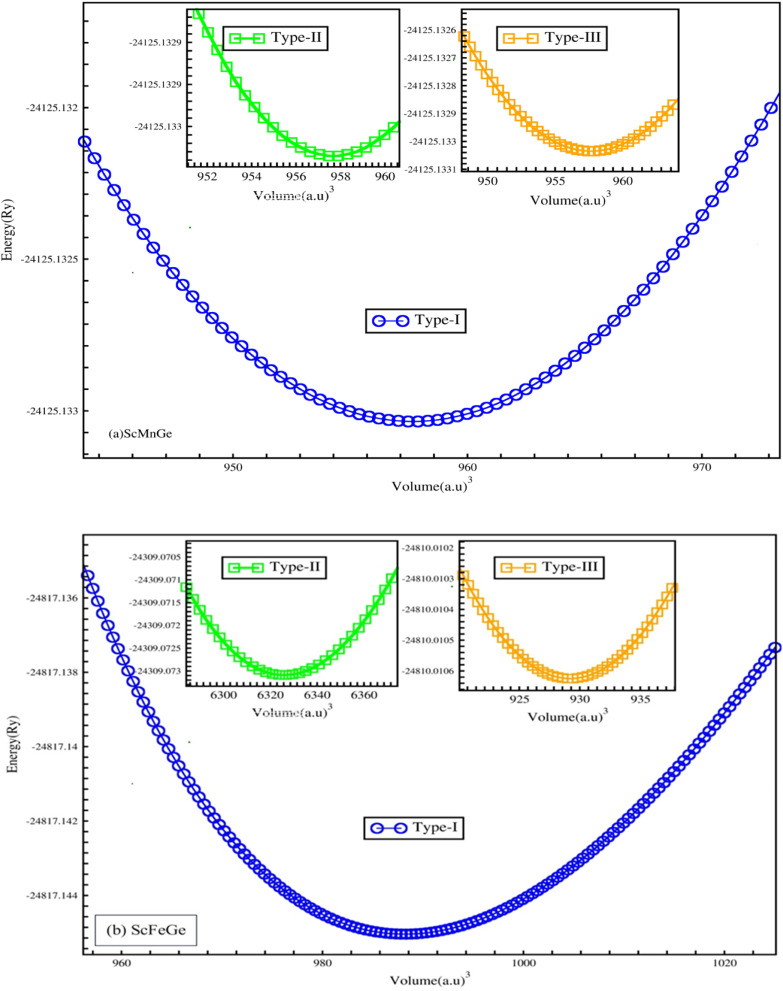
Ferromagnetic X-type-I, X-type-II, and X-type-III energy (Ry) *versus* volume (a.u.)^3^ of the (a) ScMnGe and (b) ScFeGe alloys.

**Fig. 3:**
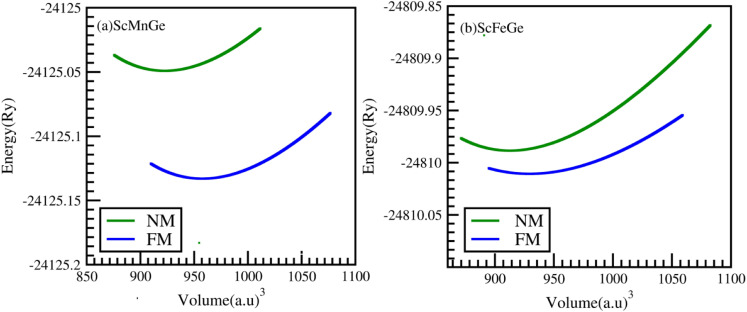
Ferromagnetic (FM) and nonmagnetic (NM) optimization plot of energy (Ry) *versus* volume (a.u.)^3^ of the (a) ScMnGe and (b) ScFeGe alloys using the GGA approximation.

ScXGe (X = Mn, Fe) compounds crystallized in the half Heusler alloys with the chemical formula XYZ.^22^ Four interpenetrating FCC sub lattices make up the unit cell; they are positioned at (0, 0, 0) and (0.5, 0.5, 0.5) for X (0.25, 0.25, 0.25) for Y, and (0.75, 0.75, 0.75) for Z. In half-Heusler compounds, the one atomic position is unoccupied. In the present work, the atomic positions (0.75, 0.75, and 0.75) are considered empty. To calculate the structural parameters for the ground state of ScXGe (X = Mn, Fe), first, the total energy *versus* volume of the unit cell was computed by means of the volume optimization process in the Birch–Murnaghan's equation of state for both the nonmagnetic (NM) and spin-polarized ferromagnetic (FM) cases. Afterward, structural properties such as lattice parameters *a* (Å), *c* (Å), bulk modulus *B* (GPa), pressure derivative of bulk modulus (*B*^p^), ground state energy (*E*_o_), and ground state volume (*V*_o_) of these ternary compounds are calculated using the volume optimization process in the Birch–Murnaghan's equation of state. The Birch–Murnaghan equation of state reveals that, during each cell optimization, energy decreases as unit cell volume increases. The term ‘ground state energy’ pertains to the minimum energy obtained, and the volume corresponding to this specific energy is referred to as the optimized volume. We have adopted three possible modes of these compounds as shown in Fig. 1, with corresponding atomic placements in the conventional cubic unit cell tabulated in Table 1.^23–28^ The optimization plots and the structural parameters of type-I, type-II, and type-III are presented in Fig. 2 and 3, and Table 2 respectively. From the ground state energies calculated in Table 2, one can state that type I is the most stable structure in both of the compounds. Furthermore, we have also optimized the NM and FM of the type-I structure. Interestingly both of the compounds have lower energies in FM than NM as shown in Fig. 3.^24,29–31^ Moreover, it was observed that by replacing the Mn atom with Fe in ScXGe (X = Mn, Fe) alloys, the lattice constants decrease to some extent which might be attributed to the lower atomic number of Mn (25) than Fe (26). The value of the bulk modulus for the alloy ScFeGe is 32.681 GPa which is lower than the ScMnGe (714.048 GPa). Smaller bulk moduli values observed in ScFeXGe alloy indicate a relatively lower resistance to external forces, suggesting that the rigidity of ScMnGe is less than that of ScFeGe. Our results indicate that the minimum energy is achieved solely at the ground state volume, revealing that all these compounds exhibit their lowest ground state energy in FM optimization. This suggests that the stability of these compounds is enhanced in the type-I FM state. It's worth noting that no prior experimental or theoretical work on the structural properties of these ScXGe (X = Mn, Fe) Heusler compounds exists, making it impossible to directly compare our findings with other existing data.

**Table 1:** Atomic occupancy Wyckoff positions XYZ for ScXGe (X = Mn, Fe) half Heusler alloys

X-Type-I	4a(0, 0, 0)	4b(½, ½, ½)	4c(¼, ¼, ¼)
X-type-II	4c(¼, ¼, ¼)	4a(0, 0, 0)	4b(½, ½, ½)
X-type-III	4b(½, ½, ½)	4c(¼, ¼, ¼)	4a(0, 0, 0)

**Table 2:** Calculated structural and total energy parameters such as lattice constants *a* (Å), *c* (Å), bulk modulus *B* (GPa), pressure derivative of bulk modulus (*B*^p^), ground state energy (*E*_o_), and ground state volume (*V*_o_) and *c*/*a* ratio of ScXGe (X = Mn, Fe) half Heusler alloys along with available experimental results for comparison

	Energy and structural properties	NM optimization	FM X-type-I	FM X-type-II	FM X-type-III	Experiment
ScMnGe	*a* (Å)	6.672	7.020	6.647	6.582	
	*c* (Å)	3.919	4.096	4.052	4.013	
	*c*/*a*	0.595	0.583	0.609	0.609	0.58349^a^
	*V*_o_	957.430	844.939	7592.362	7302.817	
	*B* (GPa)	139.062	714.048	−16.773	−27.746	
	*B* ^p^	5.228	5.582	5.000	5.528	
	*E*_o_ (Ry)	−24131.99	−33678.09	−24080.67	−22685.09	
ScFeGe	*a* (Å)	6.539	6.528	6.688	6.688	0.6118^a^
	*c* (Å)	4.143	3.979	4.078	4.078	
	*c*/*a*	0.633	0.604	0.609	0.609	
	*V*_o_	902.904	2414.931	6325.873	5684.005	
	*B* (GPa)	121.455	32.681	196.588	−16.884	
	*B* ^p^	1.854	5.000	21.139	5.000	
	*E*_o_ (Ry)	−24809.98	−34353.36	−24309.07	−24054.48	

a Ref. 6.

### 3.2 Electronic properties

**Fig. 4:**
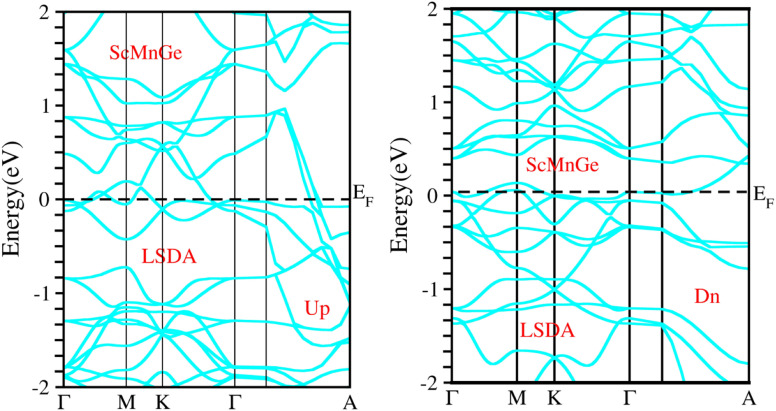
Illustration of the electronic band structure of ScMnGe alloy using LSDA approximation for spin-up and down configuration.

**Fig. 5:**
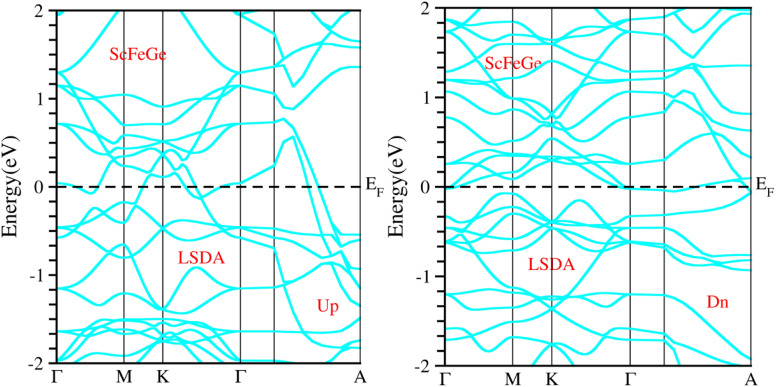
Illustration of the electronic band structure of ScFeGe alloy using LSDA approximation for spin-up and down configuration.

**Fig. 6:**
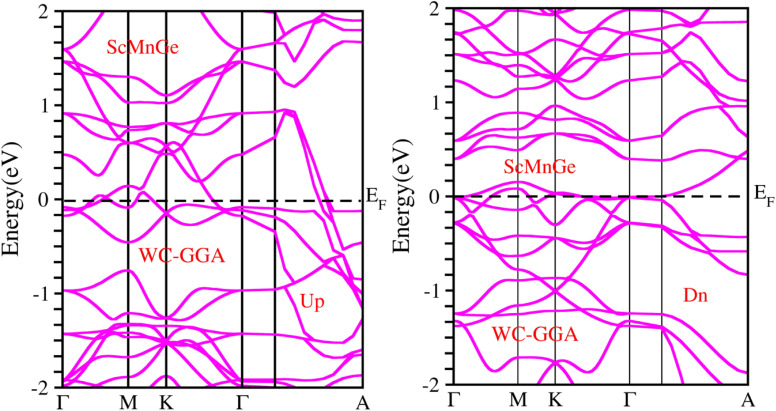
Illustration of the electronic band structure of ScMnGe alloy using WC-GGA approximation for spin-up and down configuration.

**Fig. 7:**
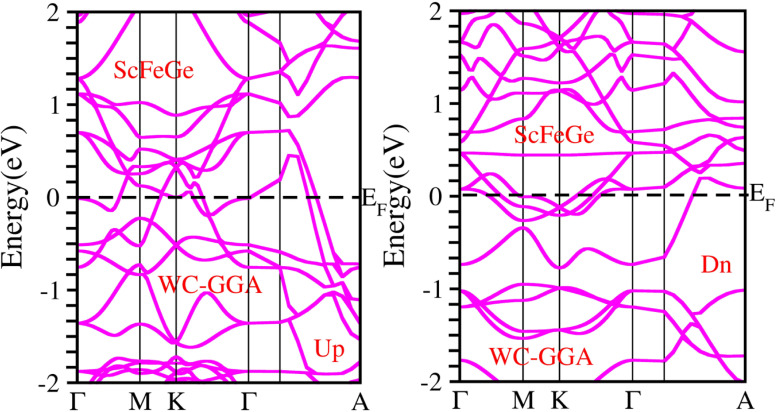
Illustration of the electronic band structure of ScFeGe alloy using WC-GGA approximation for spin-up and down configuration.

**Fig. 8:**
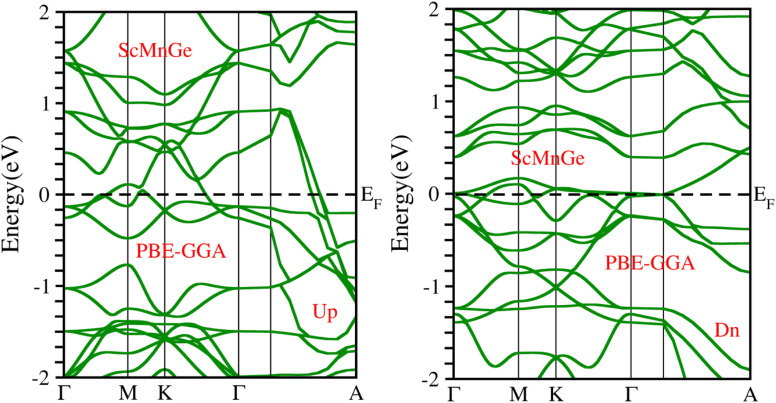
Electronic band structure of ScMnGe alloy calculated using the PBE-GGA approximation.

**Fig. 9:**
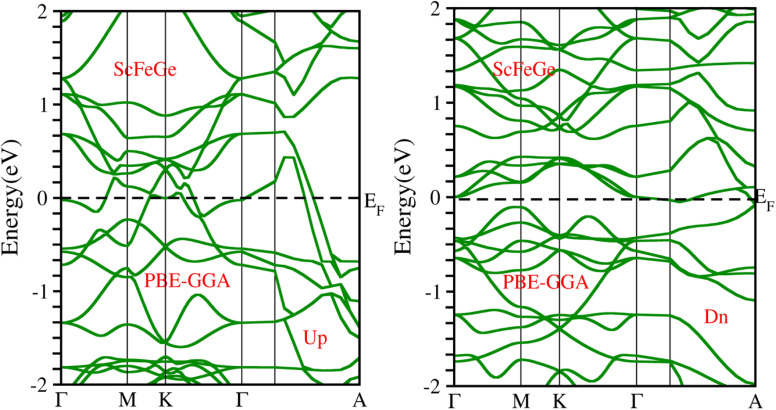
Electronic band structure of ScFeGe alloy calculated using PBE-GGA approximation.

**Fig. 10:**
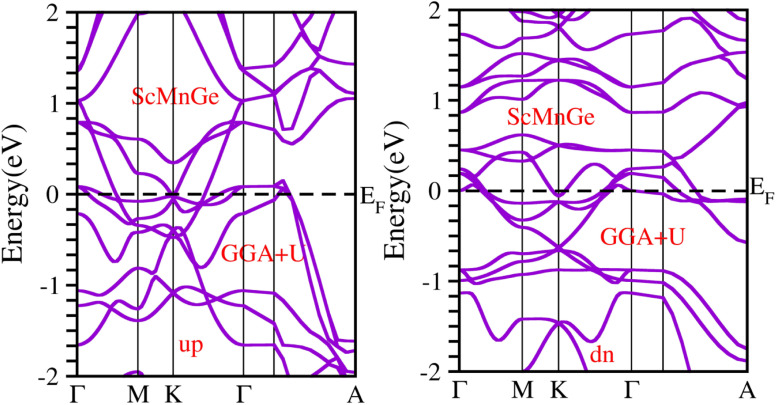
Electronic band structure of the ScMnGe alloys obtained by GGA + *U* approximation.

**Fig. 11:**
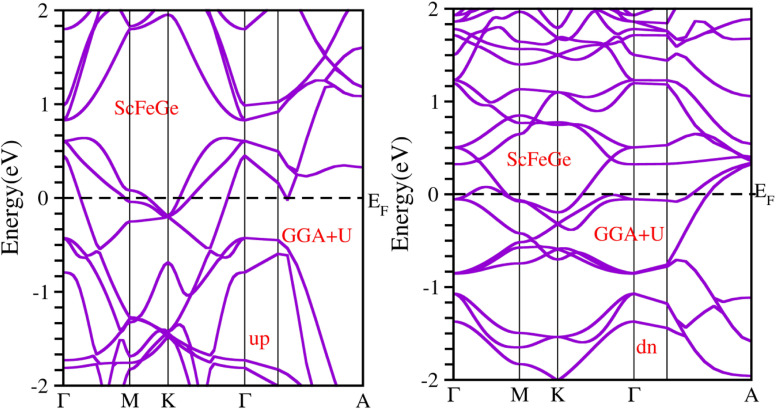
Electronic band structure of the ScMnGe alloys obtained by GGA + *U* approximation.

When considering the electronic properties, the computation of band structures plays a pivotal role in gaining insights into the nature of ScXGe (where X = Mn, Fe) Heusler compounds. In this context, the evaluation of electrical characteristics using various methods such as LSDA, WC-GGA, PBE-GGA, and GGA + *U* is of paramount importance. In our analysis, the outcomes of type-I spin-polarized ferromagnetic band structures hold particular significance in distinguishing whether a material behaves as an insulator, a metal, or a conductor. Specifically, the presence of a substantial energy gap between the conduction and valence bands (CB and VB) signifies that electrons cannot reach the Fermi level. When this energy gap is on the order of 1 electron volt (eV), a few electrons tend to breach the Fermi level and transition to the CB, resulting in a limited ability to conduct current in these compounds. In contrast, conductors feature valence electrons capable of easily crossing the Fermi level to reach the CB, essentially leading to an overlap between the valence and conduction bands. The unique characteristics of materials stem from their distinctive band structures, which consequently give rise to unconventional electrical properties when these materials are combined. In this background, we investigated the electronic properties of ScXGe (where X = Mn, Fe) Heusler compounds using self-consistent field (SCF) calculations. Fig. 4–11 present the band structures for majority and minority spin states in ScXGe (X = Mn, Fe) Heusler alloys. Specifically, Fig. 4, 6, 8 and 10 correspond to ScMnGe, while Fig. 5, 7, 9 and 11 correspond to ScFeGe. Our spin-polarized ferromagnetic calculations of type-I reveal that both the majority spin-up and minority spin-down states exhibit metallic behavior in ScXGe (X = Mn, Fe) alloys. The *U* parameters in the GGA + *U* were adopted in the range from 7 to 7.9 eV by the method introduced in ref. 21. For band structures and density of states, we have adopted 7 eV. In these materials, valence electrons within the bands traverse the Fermi level, indicating the absence of a band gap between the valence and conduction bands, thus classifying both compounds as metals.

**Fig. 12:**
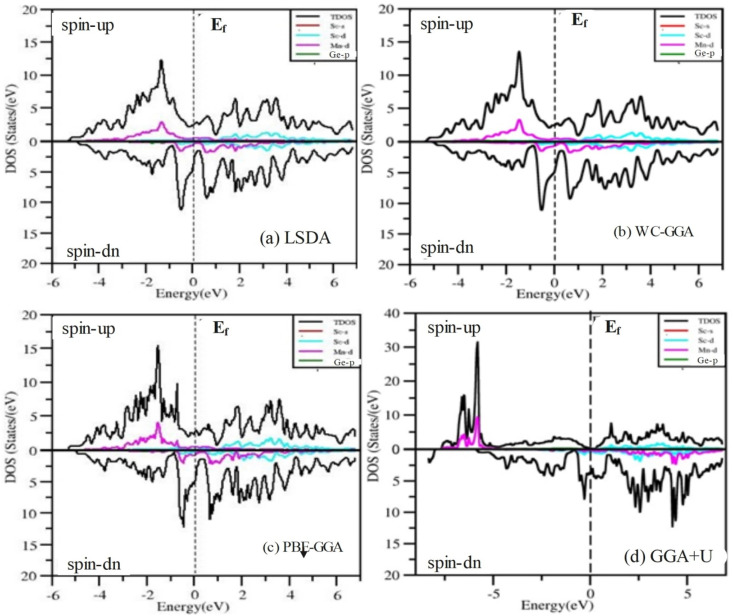
The calculated total and partial density of states ScMnGe Heusler alloys using LSDA, WC-GGA, PBE-GGA, and GGA + *U* potentials.

**Fig. 13:**
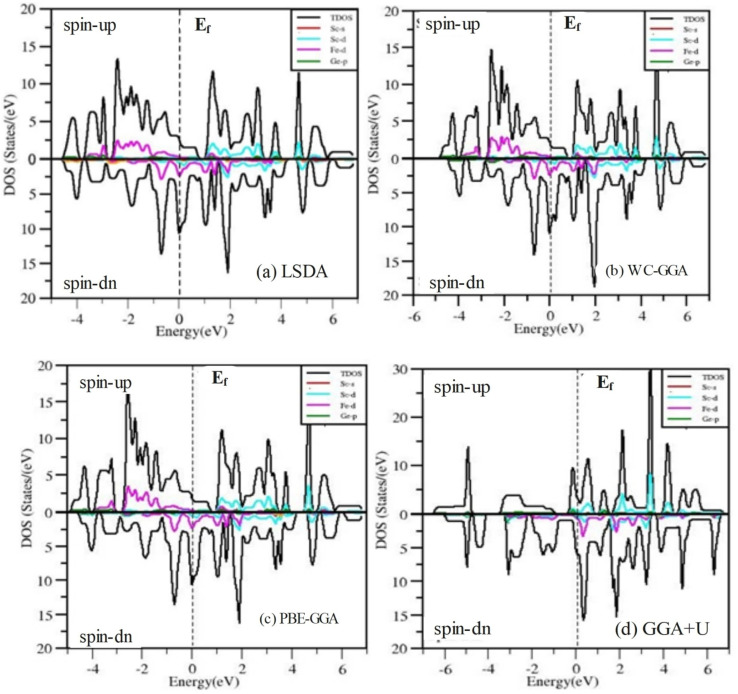
Calculated total and partial density of states of ScFeGe Heusler alloys using LSDA, WC-GGA, PBE-GGA, and GGA + *U* potentials.

To gain a deeper understanding of the electronic properties, we have analyzed the total density of states (TDOS) and partial density of states (PDOS) for the ScXGe (where X = Mn, Fe) Heusler alloys as presented in Fig. 12 and 13. In these figures, the central line, with the valence band to its left and the conduction band to its right, represents the Fermi energy (*E*_f_). Analysis of the figures leads to the following conclusions regarding the primary states in the ScXGe (where X = Mn, Fe) Heusler compounds: Sc-s, Sc-d, Mn-d, Ge-p, and Fe-d states dominate in both spin modes. Specifically, calculations indicate that Mn-d and Fe-d states make contributions that are more significant to the valence band but relatively less to the conduction band. In contrast, Sc-d states play a more substantial role in the conduction band and a lesser role in the valence band for the spin-up channel in the density of states (DOS) of ScXGe (X = Mn, Fe) Heusler alloys. For the spin-down channel, Mn-d, Fe-d, and Sc-d states contribute more to the conduction band but have relatively minor contributions to the valence band. The substitution of Mn with Fe in the ScXGe (X = Mn, Fe) Heusler alloys significantly increases the DOS at the Fermi energy in the spin-up channel. In both spin channels, Sc-d, Mn-d, and Fe-d states dominate the states in these compounds. The presence of hybridization is evident from the partial density of states (PDOS) plots, particularly the p–d hybridization showcased in Fig. 12 and 13. The increased in d-states of Mn and Fe atoms play a key role in the number of bands in the valence and conduction regions, as observed when comparing the PDOS of these alloys with their band structures. Consequently, both half-Heusler alloys, ScXGe (X = Mn, Fe), exhibit metallic characteristics.

### 3.3 Magnetic properties

**Fig. 14:**
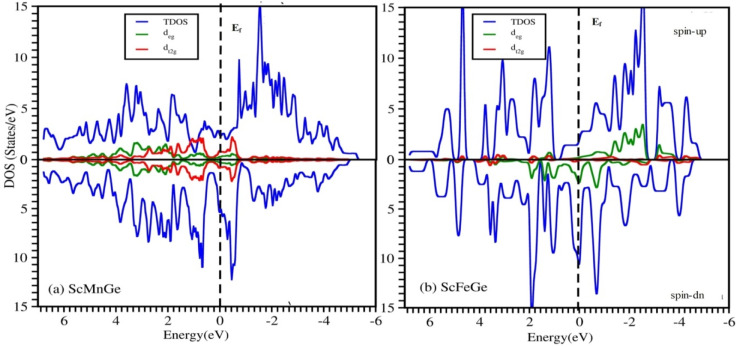
Total and partial DOS of non degenerate d_eg_ and d_t2g_ states of (a) ScMnGe and (b) ScFeGe Heusler Alloys.

The magnetic properties of ScXGe (where X = Mn, Fe) were investigated through spin-polarized type-I ferromagnetic calculations. It's important to emphasize that these investigations were conducted at absolute zero kelvin, revealing a pronounced impact on the total magnetic moment for both ScMnGe and ScFeGe. To explain the origin of the magnetic properties of these compounds, refer to the PDOS plots in Fig. 12 and 13. The Mn/Fe-d, Sc-d, and Ge-p in Fig. 12 and 13 respectively for both the compounds are responsible for the shifting of the density of states from the valence band to the conduction band, which makes the materials metallic. The PDOS of ScMnGe and ScFeGe are quite different in both the spin-up and down channels. This dissimilarity creates a shift of energy which in turn is responsible for the net magnetic moment. Moreover, the variation in positions and amplitudes of the peaks on and around the Fermi level are responsible for the shift of energy, thus creating the ferromagnetic behavior and net magnetic moment in turn. In addition, the strong Coulomb repulsion between the electron of Mn/Fe-d and Ge-p in the p–d hybridization generating crystal fields^32^ in these compounds, in which the degenerate states of Mn/Fe-d are converted into two non-degenerate states as shown in Fig. 14. The non-symmetric nature of the DOS in both spin channels predicts that ScMnGe and ScFeGe are ferromagnetic in nature. The total magnetic moment (MT) for each unit cell, along with the interstitial magnetic moment and the atomic magnetic moments of individual atoms, were calculated using various approximations including LSDA, WC-GGA, PBE-GGA, and GGA + *U*, and the results are summarized in Table 3. The negative/positive values of individual and interstitial indicate that they are antiparallel/parallel to the magnetic moments of X atoms to reduce/enhance the net magnetic moments. The inclusion of *U* parameters, which define the Coulomb interactions within the d-states and apply to all three elements, is a notable aspect. Interestingly, when GGA + *U* is employed with varying values ranging from 0.52 to 0.59, *in lieu* of LSDA, WC-GGA, and PBE-GGA, there is a notable increase in the magnetic moments of ScXGe (X = Mn, Fe) compounds. Specifically, the data indicate that Mn atoms contribute more significantly to the overall magnetic moment of ScXGe (X = Mn, Fe) compared to Fe atoms. The larger magnetic moments observed in ScXGe (X = Mn, Fe) compounds strongly suggest the presence of robust ferromagnetic behavior, signifying its potential application in spintronics devices.

**Table 3:** Magnetic moments of the mainly required interstitial region, individual atoms as well as a total cell of ScXGe (X = Mn, Fe) compounds intended for type-I ferromagnetic configurations using LSDA, WC-GGA, PBE-GGA, and GGA + *U* (*U* = 0.52, 0.53, 0.54, 0.55, 0.56, 0.57, 0.58 and 0.59 Ry)

	LSDA	WC-GGA	PBE-GGA	GGA + *U*							
				0.52	0.53	0.54	0.55	0.56	0.57	0.58	0.59
**ScMnGe alloys**											
*μ*_tot_	6.755	7.024	7.226	11.906	11.909	11.923	11.910	11.929	11.921	11.921	11.937
*μ*_int_	0.174	0.088	−0.178	−0.120	−0.133	−0.133	−0.148	−0.145	−0.160	−0.167	−0.167
*μ*_Sc_	−0.104	−0.120	−0.177	−0.310	−0.314	−0.319	−0.328	−0.332	−0.338	−0.344	−0.350
*μ*_Mn_	2.336	2.473	2.702	4.350	4.360	4.369	4.377	4.385	4.394	4.402	4.411
*μ*_Ge_	−0.026	−0.028	−0.040	−0.020	−0.021	−0.020	−0.019	−0.019	−0.018	−0.018	−0.016
*T*_c1_	1260.86	1350.11	1340.34	2220.02	2190.77	2195.45	2210.90	2185.45	2182.45	2180.45	2198.02
*T*_c2_	1245.79	1294.43	1331.00	2177.02	2178.69	2181.17	2178.87	2182.29	2180.70	2180.82	2183.61

**ScFeGe alloys**											
*μ*_tot_	6.784	7.045	6.928	9.072	11.899	11.914	11.922	11.929	11.932	11.940	11.943
*μ*_int_	0.150	−0.029	−0.052	−0.801	−0.128	−0.135	−0.140	−0.145	−0.154	−0.155	−0.161
*μ*_Sc_	0.087	−0.057	−0.071	−0.279	−0.030	−0.322	−0.327	−0.145	−0.336	−0.343	−0.349
*μ*_Fe_	2.148	2.446	2.428	3.607	4.351	4.368	4.376	4.385	4.394	4.402	4.410
*μ*_Ge_	−0.030	−0.058	−0.061	−0.003	−0.021	−0.019	−0.019	−0.018	−0.018	−0.016	−0.016
*T*_c1_	1262.09	1345.02	1260.11	1696.03	2186.01	2190.97	2198.02	2181.40	2179.80	2180.34	2199.01
*T*_c2_	1250.96	1298.31	1250.96	1656.09	2176.79	2179.57	2176.90	2181.29	2179.72	2180.17	2183.55

#### 3.3.1 Formation energy

Following the acquisition of the magnetic stable state, we proceeded to assess the thermodynamic stabilities through the calculation of the formation energy, denoted as *E*_f_^33–36^1
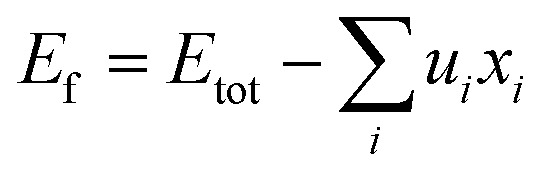
where *E*_tot_ is the calculated total energy per unit cell, *μ*_*i*_ is atomic chemical potential for element i, and the *x*_*i*_ is the quantity of element i. Formation energy is the energy needed to break the bonds separating atoms in a solid state. Table 4 summaries the computed energy of the compounds' constituent atoms as well as the formation energies of both compounds. As can be seen from Table 4 that both alloys have a negative *E*_f_, indicating them easier prepared in experiment.

**Table 4:** An overview of the formation energy *E*_f_, total energy *E*_tot_, and individual atom energies of ScXGe (X= Mn, Fe) alloys expressed in Ry

Compounds	*E*_tot_	*E*_Sc_	*E*_Mn/Fe_	*E*_Ge_	*E*_for_	*E*_coh_
**ScMnGe**						
Type-I	−24125.21	−1528.14	−2317.03	−4197.92	−16082.11	1340.17
Type-II	−24080.67	−1528.139	−2317.037	−4197.9247	−16037.569	1336.4640
Type-III	−22685.09	−1528.139	−2317.037	−4197.9247	−14641.989	1220.1657

**ScFeGe**						
Type-I	−24810.01	−1527.45	−2544.31	−4196.95	−16541.29	1378.44
Type-II	−24309.07	−1528.1391	−2545.133	−4197.927	−16037.871	1336.4892
Type-III	−24054.48	−1528.1391	−2545.133	−4197.927	−15783.281	1315.2734

#### 3.3.2 Cohesive energy

The cohesion energy (*E*_coh_), pivotal in forecasting structural stability at the ground state, quantifies the magnitude of the bonding force among atoms within materials. This cohesion energy, *E*_coh_, for these compounds, is derived using the subsequent expression,^24–28,37^2
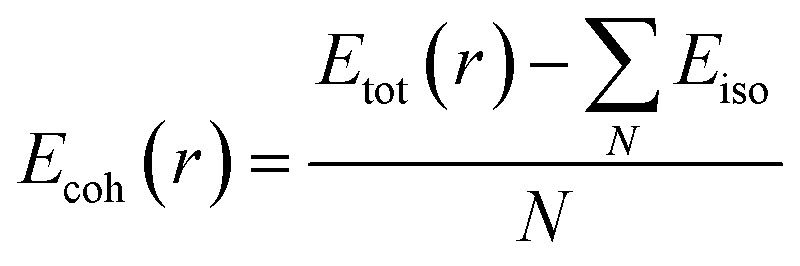
where *E*_tot_ is the equilibrium total energy per formula unit of ScXGe (X = Mn and Fe), 
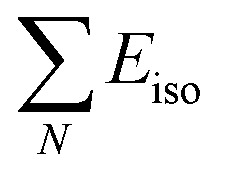
 are the sum of energies of isolated Sc, Mn, Fe, and Ge atoms respectively and N is the number of atoms in the unit cell. This is the amount of energy required to break down a crystal into fragments, and it is a measure of both the bond strengths and the mobility of the atoms within the crystal. As indicated in Table 4, when Mn is replaced by Fe the cohesive energy increases, which means that the chemical bonding in Mn-based compounds is weaker than Fe-based compounds.

#### 3.3.3 Curie temperature

The Curie temperature dictates the intensity of interaction among magnetic atoms. A higher Curie temperature signifies stronger cohesion, whereas a lower one indicates weaker interaction. The Curie temperature (*T*_c_) for ScXGe (X = Mn and Fe) compounds are computed by using the methods reported in ref. 38. The Curie temperature was evaluated using the following expression (method 1)3
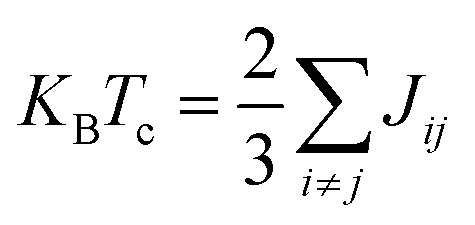
where *J*_*ij*_is the exchange interactions that can be defined in eqn (4)^28^ and *K*_B_ and represent the Boltzmann constant.4
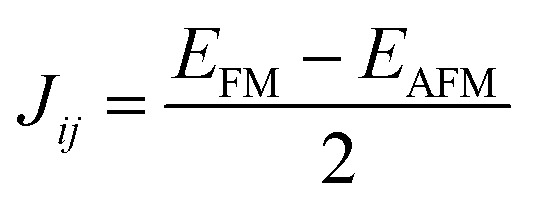
in which *E*_FM_ is the total energy of the ferromagnetic state (parallel spins) and *E*_AFM_ is that for the anti-ferromagnetic state (anti-parallel adjacent spins). The denominator 2 results from taking into account the overall energy difference between the ferromagnetic (FM) and antiferromagnetic (AFM) configurations. Eqn (3) is also called Heisenberg method. Moreover, it is also possible to determine *T*_c_ employing method 2 having in hand the total magnetic moment (*M*_tot_) using the following expression.^39–41^

5 *T*_c_ = 23 + 181*M*_tot_

The expected Curie temperatures of both compounds are shown in Table 3. Both compounds are computed at *T*_c_ values above room temperature, suggesting the possibility of their application in spintronics and magneto-electronics devices. The *T*_c_ data that was acquired for both materials shows a linear correlation between *T*_c_ and magnetic moment. When the Mn atom is replaced with Fe the total magnetic moments and hence Curie temperature decrease (see Table 3 for detail). Larger Curie temperature in Mn-based compounds shows strong ferromagnetism in Mn-based compounds as compared to Fe-based compounds.

It is important note that the structural, electronic, and magnetic properties of scandium-based half Heusler alloys ScXGe (X = Mn, Fe) were analyzed using the FPLAPW + LO method within DFT at *T* = 0 K. Due to limited computational resources, temperature-dependent studies couldn't be conducted. The calculated properties align generally with previous DFT results but slightly underestimate experimental data, likely due to the absence of lattice dynamics at *T* = 0 K, whereas experiments were conducted at room temperature. Sunil K. Karna *et al.*^19^ thoroughly examined the temperature-dependent properties of the noncentrosymmetric hexagonal ScFeGe system, including its structure, magnetism, thermodynamics, and charge transport. They prepared polycrystalline samples *via* arc melting and analyzed them using various techniques such as PXRD, EPMA, MPMS SQUID magnetometer, NPD, and XANES spectroscopy. Theoretical data from DFT calculations were compared with experimental results obtained at 2 K. Magnetic ordering was observed at *T*_N_ = 36 K (Néel temperature), along with a metamagnetic transition at 6.7 T for *H* within the hexagonal *ab* plane but not along the *c* axis. Neutron diffraction revealed an incommensurate helimagnetically ordered state below *T*_N_ with a wave vector *k* = (0 0 0.193) and a magnetic moment of *μ*_S_ = 0.53*μ*_B_/Fe aligned within the *ab* plane. Unusual magnetoresistance behavior was observed below *T*_N_, including positive MR (magnetoresistance) below *T*_N_ and at fields *H* < *H*_MM_ for *H* perpendicular to the *c* axis.

## 4. Conclusion

In summary, we have explored the structural, electrical, and magnetic properties of ScXGe (X = Mn, Fe) compounds by using first-principles calculations based on density functional theory (DFT). All ScXGe (where X = Mn, Fe) Heusler alloys have the lowest ground state energy when it comes to spin-polarized optimization, according to structural parameter optimizations carried out using both spin-polarized and non-spin-polarized methods. For both the studied alloys, the ferromagnetic state becomes more energetically stable. The compounds are found to be metallic in nature and the compound's TDOS and PDOS outcomes are consistent with the band structure's result. Further, the p–d hybridization of each compound is also confirmed by the PDOSs. Magnetic properties indicate a significant enhancement in the magnetic moments of ScXGe (X = Mn, Fe) alloys when GGA + *U* is utilized in place of LSDA, WC-GGA, and PBE-GGA. Particularly, Mn atoms play a more substantial role in contributing to the overall magnetic moment compared to Fe atoms. The negative values for formation energy (−16082.11 for ScMnGe and −16541.29 for ScFeGe) underscore the thermodynamic stability of these compounds and the presence of strong atom-to-atom bonds. Moreover, substituting Mn with Fe in the ScXGe (X = Mn and Fe) alloys leads to a reduction in cohesive energy, indicating that the chemical bonding in Mn-based compounds is stronger than in Fe-based compounds. The Curie temperatures for ScXGe (X = Mn, Fe) are notably different, with values of 2177.023 K and 1656.099 K, respectively, suggesting that Mn-based compounds exhibit stronger ferromagnetism compared to their Fe-based counterparts due to the higher Curie temperature. The intriguing structural, electronic, and magnetic characteristics of ScXGe (X = Mn, Fe) half-Heusler alloys explored in this work highlight their potential importance in the field of spintronic devices. As a result, our research provides a strong basis for future experimental verification.

## Data availability

The data used in this work can be available on reasonable request to the corresponding author.

## Conflicts of interest

There are no conflicts to declare.

## Supplementary Material
